# S2*-ODM: Dual-Stage Improved PointPillar Feature-Based 3D Object Detection Method for Autonomous Driving

**DOI:** 10.3390/s25051581

**Published:** 2025-03-04

**Authors:** Chen Hua, Xiaokun Zheng, Xinkai Kuang, Wencheng Zhang, Chunmao Jiang, Ziyu Chen, Biao Yu

**Affiliations:** 1 School of Electrical Information Engineering, Changzhou Institute of Technology, Changzhou 213031, China; ba20168187@mail.ustc.edu.cn (C.H.); 22021534@czu.cn (W.Z.); 2 Hefei Institutes of Physical Science, Chinese Academy of Sciences, Hefei 230031, China; kxk296097205@mail.ustc.edu.cn (X.K.); sa252@mail.ustc.edu.cn (C.J.); chenzy1831@mail.ustc.edu.cn (Z.C.); byu@hfcas.ac.cn (B.Y.); 3 Hunan Vanguard Group Corporation Limited, Changsha 410100, China; 4 Science Island Branch, University of Science and Technology of China, Hefei 230026, China

**Keywords:** point cloud, autonomous driving, 3D object detection, PointPillar, LiDAR

## Abstract

Three-dimensional (3D) object detection is crucial for autonomous driving, yet current PointPillar feature-based methods face challenges like under-segmentation, overlapping, and false detection, particularly in occluded scenarios. This paper presents a novel dual-stage improved PointPillar feature-based 3D object detection method (S2*-ODM) specifically designed to address these issues. The first innovation is the introduction of a dual-stage pillar feature encoding (S2-PFE) module, which effectively integrates both inter-pillar and intra-pillar relational features. This enhancement significantly improves the recognition of local structures and global distributions, enabling better differentiation of objects in occluded or overlapping environments. As a result, it reduces problems such as under-segmentation and false positives. The second key improvement is the incorporation of an attention mechanism within the backbone network, which refines feature extraction by emphasizing critical features in pseudo-images and suppressing irrelevant ones. This mechanism strengthens the network’s ability to focus on essential object details. Experimental results on the KITTI dataset show that the proposed method outperforms the baseline, achieving notable improvements in detection accuracy, with average precision for 3D detection of cars, pedestrians, and cyclists increasing by 1.04%, 2.17%, and 3.72%, respectively. These innovations make S2*-ODM a significant advancement in enhancing the accuracy and reliability of 3D object detection for autonomous driving.

## 1. Introduction

In autonomous driving (AD), the ability to detect objects in three dimensions (3D) is paramount for ensuring safe navigation. LiDAR technology, which generates 3D point clouds, provides autonomous vehicles with detailed spatial data that help identify and locate nearby objects such as vehicles, pedestrians, and cyclists [1,2]. This ability to capture and process 3D data offers a distinct advantage over traditional 2D image-based methods, as 3D detection is far better equipped to address challenges like occlusion and object overlap, which are common in real-world driving scenarios. These challenges can severely hinder 2D systems, but 3D detection methods can improve the systems’ decision-making and path-planning capabilities by providing more accurate and complete information about the environment. This enhanced situational awareness is crucial for safe navigation and for making the real-time decisions required in autonomous driving (AD).

However, working with LiDAR point cloud data introduces several challenges. The data are often unordered, sparse, and lacking the structured grid format found in traditional image data, making it difficult to extract useful features directly. In addition, the sparse nature of point clouds means that much of the data can be distributed unevenly across the scene, leading to incomplete representations of the environment. This sparsity makes it necessary to use advanced algorithms and deep learning-based methods for feature extraction [3,4], which can better handle the complexity and irregularity of point cloud data. Furthermore, detecting and segmenting objects accurately in environments with occlusions, variable lighting, or adverse weather conditions remain a significant hurdle [5]. In these cases, traditional methods may fail to capture the full extent of objects or may confuse multiple objects due to incomplete or noisy data. As a result, developing precise and reliable 3D object detection methods is essential for the continued advancement of AD technology and for ensuring that autonomous systems can operate safely and effectively in diverse and challenging environments.

To address these challenges, various techniques have been employed, including deep learning-based approaches [6,7] for automatic feature extraction, which have significantly enhanced detection performance. These methods enable the network to learn complex representations of the environment without requiring extensive manual feature engineering. Furthermore, to overcome the sparsity inherent in point cloud data, methods like voxelization and hierarchical sampling have been developed. These techniques convert point cloud data into more structured forms, such as 3D voxel grids and hierarchical structures, which are easier to process and analyze. These innovations have made 3D object detection methods increasingly effective for practical AD applications by improving detection accuracy, enabling faster processing times, and reducing the computational load.

Three-dimensional object detection techniques can be broadly classified into three main categories: range image-based, point-based, and voxel-based approaches [8]. Range image-based techniques utilize depth images generated by LiDAR or depth cameras, converting 3D point cloud data into 2D depth maps for feature extraction through conventional image processing methods. While these approaches offer computational efficiency, especially in handling large point clouds in complex scenes, they often suffer from issues like sparse depth data. This is particularly problematic at greater distances or with reduced resolution, which can significantly impact detection accuracy [9,10]. In these cases, the depth maps may fail to capture critical object details, especially when objects are far away or partially occluded, reducing the overall effectiveness of the detection system.

On the other hand, point-based methods, such as PointNet [11], process unordered point cloud data directly by analyzing the spatial positions and local structures of individual points. This approach preserves more detailed information and avoids the loss of data that can occur in range image-based methods. However, point-based methods struggle with occlusions and object overlaps, which are common in dynamic environments. The unordered nature of point clouds makes it difficult to infer the relationships between points in complex settings, and the approach is highly sensitive to point cloud sparsity. PointNet++ [12], an improved version, addresses some of these challenges by incorporating hierarchical feature learning, enabling the network to focus on local features in a more structured way. However, this improvement comes at the cost of increased computational complexity, as large amounts of point cloud data need to be processed to extract the relevant features.

Voxel-based methods discretize point cloud data into 3D voxel grids, transforming sparse data into a more structured form that is easier to process and analyze [13]. The pillar-based approach optimizes 3D volumetric grid processing by projecting data onto 2D planes through vertical axis elimination, reducing computational demands but compromising the retention of spatial hierarchies (e.g., vertical element distribution and positional relationships [14]. This approach improves processing efficiency, allowing for faster detection. However, it can lead to the loss of critical point cloud information, such as the precise spatial location and relative positions of points within each voxel, which may impact the accuracy of the detection. For example, the PointPillar method [15] uses pillar-based feature aggregation for detection, converting point cloud data into vertical pillars. While this method improves processing efficiency and allows for real-time detection, it struggles to accurately represent the relationships between the pillars and the full point cloud. This limitation is particularly problematic in scenarios involving occlusions or overlaps, where the pillars representing different objects may be incorrectly grouped together [16].

These issues often lead to under-segmentation, where pillars from different objects are merged into a single group or missed detections due to the failure to properly separate overlapping objects. Furthermore, variability in pillar-to-point cloud correspondence across different datasets or scenarios can introduce instability in the detection results [17]. In such cases, the model may fail to consistently detect or localize objects, resulting in false positives or missed detections. These challenges emphasize the need for more advanced techniques that can better account for the intricate relationships between pillars and ensure accurate segmentation, even in complex and occluded environments.

In conclusion, while each of the aforementioned approaches has its own strengths and weaknesses, it is clear that a combination of techniques, such as the use of deep learning-based feature extraction and methods to handle point cloud sparsity, is necessary for achieving high-precision 3D object detection in autonomous driving applications. The ongoing development of methods that better capture both the local and global relationships in point cloud data is crucial for improving the robustness and accuracy of AD systems, allowing them to handle the complexities of real-world driving environments.

This paper proposes a dual-stage enhanced PointPillar feature-based 3D object detection method (S2*-ODM) to overcome the limitations of existing detection techniques. The method consists of three main components: the pillar feature encoding module, the backbone network module, and the detection head module. Significant improvements were made to both the pillar feature encoding and the backbone network modules, with the following key contributions:

- To address the under-segmentation issue arising from feature omissions, this paper introduces a dual-stage pillar feature encoding (S2-PFE) module. S2-PFE captures the intricate physical relationships both within and between pillars, enhancing object differentiation and mitigating under-segmentation problems in occluded and overlapping scenes.
- To address the challenge of insufficient key feature extraction in object detection, this paper presents an enhanced network backbone structure that integrates SeNet. This integration improves the extraction of critical features from pseudo-images while suppressing irrelevant information.

The structure of this paper is as follows. Section 2 introduces the related work. Section 3 introduces the PointPillar-based 3D object detection framework. Section 4 discusses the dual-stage PointPillar feature encoding module. Section 5 focuses on the Setnet improved backbone network, while Section 6 focuses on experiments and analyses. Finally, Section 7 provides our conclusions and future work.

## 2. Related Work

Voxel-based 3D detection methods are commonly utilized in autonomous driving systems by transforming point cloud data into a 3D grid of voxels, where each voxel represents information from a specific region in space. This transformation enables the use of convolutional neural networks (CNNs) to extract meaningful features from the voxelized data, facilitating efficient object detection [18]. For example, VoxelNet [13] introduced voxel feature encoding (VFE) and 3D convolutional layers, which are pivotal for extracting features from voxelized data, allowing the network to understand the spatial structure and local relationships within the point cloud. However, one of the main challenges with voxel-based methods is the high number of voxels, especially in complex environments. This results in considerable computational overhead, which can slow down both training and inference, limiting the scalability of the method for real-time applications in autonomous driving.

To mitigate these challenges, methods like SECOND [19] have employed sparse 3D convolution, which focuses only on non-empty voxels, thus ignoring empty regions in the point cloud and significantly speeding up both training and inference. This approach improves efficiency but can present difficulties when deployed in real-world settings, where data density and the distribution of points can vary dramatically. In contrast, PointPillars [15] simplifies the voxelization process by projecting point clouds onto a 2D plane and encoding voxel data as pixel values in a pseudo-image. This projection reduces the computational complexity of processing the point cloud, making it more efficient and enabling faster detection. PointPillars provides a more accessible solution without sacrificing the accuracy of the system, especially for larger objects.

Another approach, CenterPoint [20], further refines the process by predicting object properties at voxel centers and using 2D convolutional networks for local feature extraction. This method strikes a balance between accuracy and speed, particularly for detecting smaller objects. By focusing on voxel centers and simplifying the computation, CenterPoint allows for real-time detection of both large and small objects, making it well-suited for various autonomous driving applications. Despite these advancements, voxel/pillar-based methods still face challenges, such as potential information loss due to uniform voxelization, which can lead to uneven sampling and a decrease in the quality of detected features [21]. Additionally, these methods often have high false and missed detection rates for small or dense objects, which are common in real-world environments where objects vary in size and shape [22]. Under-segmentation is also a concern, as the neglect of local-global feature relationships can cause objects to be incorrectly segmented, leading to inaccurate detection and localization results [23].

Several improvements have been introduced to address these limitations. For instance, refined voxelization techniques have been proposed to achieve better sampling and minimize information loss. Additionally, attention mechanisms have been integrated into 3D object detection pipelines to refine feature extraction and boost performance. Techniques like SCNet [24] improve segmentation by subdividing grids, which leads to more accurate boundaries and object delineation. SA-Det3D [25], another notable improvement, combines convolutional and self-attention features, resulting in enhanced performance, particularly in complex scenes with occlusions or overlapping objects. Moreover, PV-RCNN [26] merges voxel and point cloud features, allowing it to handle a variety of object sizes and shapes more effectively, thereby improving detection accuracy. Compared with PointNet++, SA-Det3D, and PV-RCNN, the innovation of this paper lies in its unique dual-stage design. In the first stage, local MLPs are used to encode the geometric features within individual pillars, thereby efficiently extracting local information. In the second stage, sparse convolution is employed to model the topological relationships across pillars, such as the geometric consistency between adjacent pillars, thereby achieving the integration of global information. Additionally, in terms of lightweight improvements, pillar pruning is employed to reduce redundant computations, thereby enhancing computational efficiency. This stands in stark contrast to the dense point processing approach used in PointNet++.

Attention mechanisms have found wide application in computer vision tasks, including classification, detection, and segmentation [27], due to their ability to help networks focus on the most relevant features, thereby improving detection accuracy [28]. These mechanisms are particularly beneficial in point cloud data detection. Spatial attention allows the model to prioritize critical information from specific regions of the point cloud, enhancing the network’s ability to detect objects that might be otherwise obscured or difficult to differentiate. Similarly, channel attention emphasizes the importance of features from specific channels, ensuring that the network focuses on the most important attributes, such as object boundaries and fine-grained details [29]. In the context of 3D object detection, self-attention [30] and transformer-based models [31] have also been explored to capture global dependencies within the point cloud. However, research suggests that self-attention modules may not be as effective for 3D point cloud data as other mechanisms like Squeeze-and-Excitation (SE) [32] and Convolutional Block Attention Module (CBAM) [33], which have been demonstrated to significantly enhance the refinement of 3D point cloud features.

Li et al. [34] introduced three enhanced attention mechanisms—SOCA, SOPA, and SAPI—which are specifically designed to improve feature extraction in 3D object detection. These attention mechanisms help the model focus on key spatial regions, improving the network’s ability to detect objects even in complex environments. Furthermore, self-attention variants like FSA (Feature-wise Self-Attention) and DSA (Dynamic Self-Attention) are employed to improve contextual modeling by combining convolutional features with self-attention features. These variants are particularly useful in modeling long-range dependencies in point clouds, which is crucial for understanding the relationships between objects in complex and cluttered environments. Additionally, attention mechanisms have proven highly beneficial for point cloud segmentation, where networks based on point attention mechanisms have led to significant improvements in segmentation performance [35]. By applying these techniques, 3D object detection systems can achieve more precise and reliable results, improving the overall safety and performance of autonomous driving systems.

In summary, while voxel-based methods have demonstrated significant progress in 3D object detection for autonomous driving, their limitations in terms of computational overhead, information loss, and detection accuracy for smaller or occluded objects highlight the need for further improvements. The integration of attention mechanisms, refined voxelization strategies, and hybrid approaches that combine voxel and point cloud features offer promising solutions to these challenges, advancing the field of 3D object detection and enhancing the effectiveness of autonomous driving systems in real-world scenarios.

## 3. PointPillar-Based 3D Object Detection Framework

**Figure 1:**
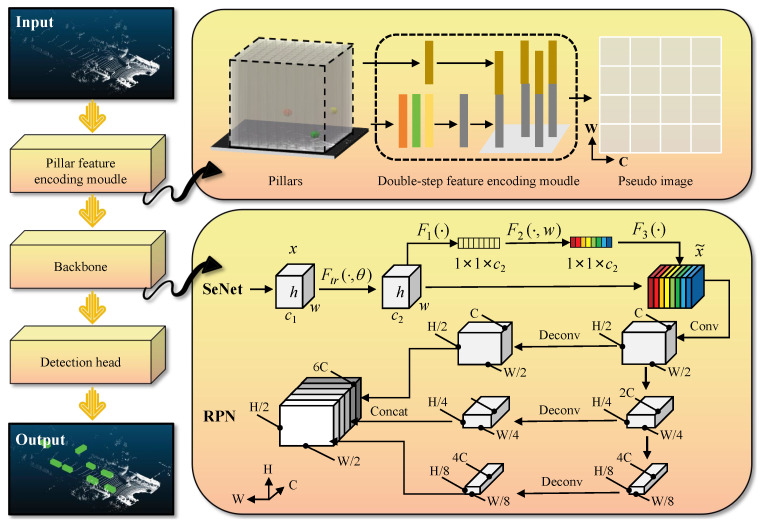
Framework of the dual-stage improved PointPillar feature-based 3D object detection method (S2*-ODM). The overall architecture of the S2*-ODM framework consists of four key modules: **(1) Point Cloud Input Layer:** receives raw LiDAR data, including coordinates and reflection intensity. **(2) Dual-stage Feature Encoding (S2-PFE) Module:** —*Stage I (Local):* extracts geometric features from individual pillars using an MLP. —*Stage II (Global):* aggregates neighboring pillar features via sparse convolution and fuses them into enhanced features. **(3) SeNet Backbone:** applies channel-wise attention to the pseudo-image tensor, emphasizing critical feature channels. **(4) Detection Head:** predicts 3D bounding box parameters from multi-scale features.

This paper introduces an innovative and highly effective approach to 3D object detection, leveraging cylindrical representations to address the complexities of accurately detecting objects in three-dimensional space. The overall framework of this novel method is illustrated in Figure 1, which provides a comprehensive overview of the detection pipeline. The detection process consists of three core components: a cylindrical feature encoding module, a backbone network module, and a detection head responsible for the final prediction of 3D bounding boxes.

The method begins by transforming the raw, unordered point cloud data into a structured cylindrical form. This transformation is achieved by partitioning the point cloud into regular 2D grid cells, each of which corresponds to a cylindrical unit. This structuring step is crucial as it enables the network to work with a regular and organized representation of the data, making it easier to process compared to raw, unstructured point clouds. Once this transformation is completed, a convolutional neural network (CNN) is employed to extract informative features within each cylindrical unit. The features learned from these cylindrical units are then mapped onto a 2D pseudo-image, which serves as a 2D representation of the cylindrical features. This pseudo-image acts as a bridge, allowing the 2D convolutional network to handle the previously 3D data in a way that simplifies the subsequent learning process.

The backbone network module, which integrates a Region Proposal Network (RPN) and a Squeeze-and-Excitation Network (SeNet), then extracts high-level features from this 2D pseudo-image. The RPN component is responsible for generating candidate regions that could potentially contain objects, while the SeNet dynamically adjusts the importance of different feature channels, helping the network focus on the most relevant information for object detection. These enhanced features are then passed on to the detection head, which is responsible for predicting the 3D bounding boxes. The bounding box predictions include the object’s precise location, size, and orientation, which are crucial for accurate object detection in real-world environments, such as in autonomous driving applications.

By using this method, the framework is able to achieve precise and reliable 3D object detection, even in challenging scenarios where objects are located at various distances, angles, and orientations. The cylindrical representation allows for more efficient feature extraction and better handling of point cloud data, making the method particularly suitable for real-time applications in autonomous driving, where accurate and fast 3D detection is essential for safe navigation in complex environments. Through this approach, the paper contributes to advancing the field of 3D object detection by providing a robust solution that combines the benefits of structured representation, convolutional feature learning, and advanced detection techniques.

## 4. Dual-Stage Pillar Feature Encoding Module

Traditional voxel and pillar encoding techniques have revolutionized the way point cloud data are processed by dividing the data into discrete, organized structures, such as voxels or pillars. These techniques work by grouping the raw point cloud data into manageable sections, where the features within each defined structure are aggregated. This approach significantly reduces computational complexity and enhances processing speed, making it suitable for real-time applications such as autonomous driving and robotics. By breaking down the data into smaller, more structured units, these methods enable efficient feature extraction and can deliver quick results for local details within each voxel or pillar.

Despite these notable advantages, traditional voxel and pillar encoding methods are not without their limitations. While they excel in capturing the local features within each voxel or pillar, they often fail to effectively represent the global properties of the overall point cloud. Specifically, these methods struggle to capture the intricate relationships between the individual pillars or voxels and the broader context of the entire point cloud. As a result, they may miss important global patterns or fail to account for the interdependencies between the different parts of the point cloud. This limitation becomes particularly evident in complex or occluded scenes, where the relationships between distant objects or overlapping structures are critical for accurate object detection. In such scenarios, the lack of a holistic understanding of the point cloud data can severely hinder the performance of detection models, especially when precise contextual awareness is needed for proper object localization and classification.

**Figure 2:**
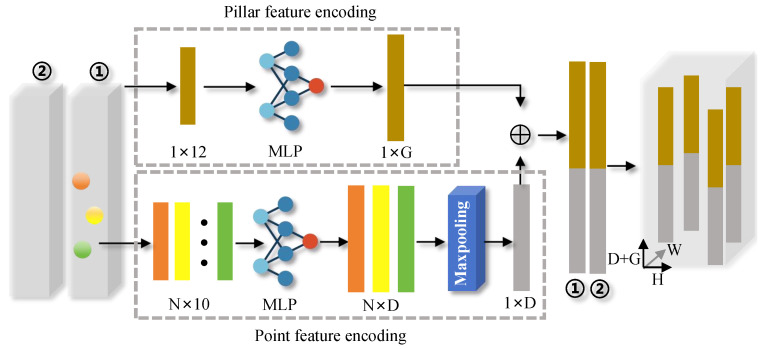
The dual-stage pillar feature encoding (S2-PFE) module framework.

To address this challenge, this paper introduces the S2-PFE module, which is integrated into the pillar-based detection framework. The S2-PFE module is designed to enhance the model’s ability to understand both the local structural features of individual pillars and the global distribution of pillars across the entire point cloud. By considering the relationships both within each pillar and between multiple pillars, the S2-PFE module overcomes the limitations of traditional methods by fostering a more holistic understanding of the point cloud data. As illustrated in Figure 2, the S2-PFE module consists of three key components, each of which contributes to improving the detection capabilities of the model:

(1) Point Feature Encoding: This component is responsible for aggregating and extracting features from each point in the point cloud. By capturing fine-grained information about the points, this step ensures that the model has a comprehensive understanding of the local characteristics of the data, including fine details that are critical for precise detection.

(2) Pillar Feature Encoding: This module focuses on extracting features at the pillar level, which allows the model to capture broader, more spatially structured patterns across the point cloud. The pillar-level encoding facilitates the representation of larger-scale features and ensures that the model has an understanding of the global structure of the point cloud, which is crucial for object detection in more complex scenes.

(3) Feature Fusion: After the point and pillar features are extracted, this component merges the two types of features to generate more comprehensive and effective representations. By combining local and global features, the feature fusion step enables the model to leverage both the detailed information at the point level and the contextual information at the pillar level. This fusion process results in more robust and accurate features that better capture the underlying relationships within the point cloud.

Together, these components enable the S2-PFE module to provide a more holistic representation of the point cloud data, improving the model’s ability to detect objects in diverse and complex environments. By incorporating both local and global perspectives, the S2-PFE module enhances the overall detection performance, ensuring that the model can handle challenging scenarios, such as occlusion, varying object scales, and intricate spatial relationships between objects. Through this novel approach, the S2-PFE module makes a significant contribution to the advancement of pillar-based 3D object detection, offering a more effective solution for real-world applications.

To enhance the efficiency of point cloud data processing, the S2-PFE module defines each point’s coordinates along the X-, Y-, and Z-axes as $x_{i}$, $y_{i}$, and $z_{i}$, respectively. To streamline the data transformation, the Z-axis information is omitted, effectively creating pillars of a fixed height *H* along the Z-axis. The point cloud is then partitioned into pillar units along the X- and Y-axes, with dimensions $L_{c}$ and $L_{w}$, respectively. Each pillar unit is represented as $\xi_{i}={\left[x_{i},y_{i},z_{i},\eta_{i}\right]}^{T}$, $\xi_{i}\in R^{4}$ and $\eta_{i}$ represents the laser reflectivity value of the point (laser reflectivity is a property of the point cloud captured by the LiDAR sensor, characterizing the intensity of the reflection of laser pulses from the target surface). By excluding the Z-axis data, the point cloud is transformed into a simplified 2D pillar structure. This approach reduces the complexity of processing the data while significantly enhancing the efficiency of handling point cloud data, making it more suitable for real-time applications in autonomous driving.

### 4.1. Point Feature Encoding Method

The point feature encoding method presented in this paper aggregates individual point features to represent the corresponding pillar features more effectively. Initially, to capture a more comprehensive set of characteristics for each point, the original 4D feature vector is expanded into a higher-dimensional vector. This expanded vector is denoted as $\xi_{i}^{n}={\left[\xi_{i},\psi_{i},\phi_{i}\right]}^{T},\xi_{i}^{n}\in R^{10\times N}$, where $\psi_{i}\in R^{3}$ represents the distance from the point to the arithmetic mean position of all points within the pillar along the X-Y-Z directions. Similarly, the offset $\phi_{i}\in R^{3}$ indicates the displacement of each point relative to the center of the pillar. These enhanced feature vectors are then processed further through a multi-layer perceptron (MLP) network. The MLP maps the features into a higher-dimensional space, improving the ability of the model to represent complex point characteristics. This process significantly enhances the feature representation capabilities, enabling more accurate pillar-level feature encoding for subsequent tasks:

(1) $$\begin{array}{c} \xi_{i}^{m}=G\left(\xi_{i}^{n},W_{m},B_{m}\right) \end{array}$$

here, $\xi_{i}^{m}\in R^{D\times N}$ refers to the features of each point. G(·) represents a stack of MLP, each comprising a Batch Normalization (BN) layer and a Rectified Linear Unit (ReLU) activation layer. The term $W_{m}$ denotes the learned weights, while $B_{m}$ represents the bias values. Subsequently, a max pooling operation $P_{\max}\left\{\cdot \right\}$ is applied to aggregate the features of all points within pillar *j*, resulting in a single feature vector that encapsulates all the information of that pillar. This feature vector $\xi_{j}^{m}$ is expressed as follows:

(2) $$\begin{array}{c} \xi_{j}^{m}=P_{\max}\left\{\xi_{i}^{m}\right\},\xi_{j}^{m}\in R^{D} \end{array}$$

### 4.2. Pillar Feature Encoding Method

In 3D object detection, simply aggregating pillar features from point clouds is often not enough to capture the complex geometric relationships and global context necessary for accurate detection. To overcome this limitation and improve system performance, this paper introduces a second-stage feature encoding mechanism, known as the Pillar Feature Encoding Unit. This unit is specifically designed to encode not only the intrinsic features of the individual pillars but also their relationships with the broader point cloud. By considering both local pillar-level characteristics and their connections within the entire scene, this unit provides a more comprehensive representation, which significantly enhances the model’s ability to discern complex patterns and improve detection accuracy in challenging environments.

First, the arithmetic mean of all points within pillar *j*, denoted as $\chi_{j}^{c}\in R^{3}$, represents the centroid of the pillar, while the central value $\chi_{j}^{p}\in R^{3}$ of pillar *j* serves as the center of the pillar. Next, the arithmetic mean ${\bar{\chi }}_{\Theta }^{c}\in R^{3}$ of all points is computed to represent the centroid of the entire point cloud Θ, and the coordinates ${\bar{\chi }}_{\Theta }^{p}\in R^{3}$ are considered the center coordinates of the point cloud Θ. The offset $\Delta \chi_{j}^{c}=\chi_{j}^{c}-{\bar{\chi }}_{\Theta }^{c}$ from each pillar to the Θ centroid and the offset $\Delta \chi_{j}^{p}=\chi_{j}^{p}-{\bar{\chi }}_{\Theta }^{p}$ from each pillar to the Θ center are then calculated. At this stage, the following features $\delta_{j}^{n}$ are obtained:

(3) $$\begin{array}{c} \delta_{j}^{n}={\left[\chi_{j}^{c},\chi_{j}^{p},\Delta \chi_{j}^{c},\Delta \chi_{j}^{p}\right]}^{T}\in R^{12}. \end{array}$$

These features are then expanded and transformed into a higher-dimensional space using a multi-layer perceptron (MLP) network. This transformation enables the network to capture more complex patterns and relationships within the data, thereby improving the feature representation capabilities. The process is expressed as follows:

(4) $$\begin{array}{c} \delta_{j}^{m}=G\left(\delta_{j}^{n},W_{m},B_{m}\right) \end{array}$$

where $\delta_{j}^{m}$ represents the pillar features, which encompasses not only the characteristics of the pillar itself but also the relationships between pillars and the global features of the entire point cloud.

### 4.3. PointPillar Dual Feature Fusion

In the 3D object detection approach presented in this paper, the process of dual feature fusion for point pillars involves integrating features from multiple sources to create a richer and more detailed representation of pillar characteristics. These features are divided into two main types: the first type is extracted from individual points within the point cloud, while the second is derived from the pillar-level features themselves. By combining these two feature types, which capture important details both at the local and global scales, we can achieve a more accurate description of the overall pillar characteristics. The feature fusion process in this module can be mathematically represented by the following formula:

(5) $$\begin{array}{c} U_{j}^{c}=F\left(\xi_{j}^{m},\delta_{j}^{m}\right) \end{array}$$

Here, F represents the feature concatenation function, resulting in the fused pillar features. After obtaining the fused features, each pillar is mapped to a plane based on its coordinates to produce a pseudo-image M with dimensions H×W×(D+G). For simplicity, (D+G) is denoted as *C*.

After generating the pseudo-image, the next step is to extract relevant features using a convolutional neural network (CNN). To enhance the backbone network’s performance, the SeNet is incorporated. The SeNet is designed to help the network focus on critical information, enhancing feature extraction by suppressing irrelevant details. Once SeNet processes the features, the backbone network connects to a region proposal network (RPN). The RPN is a dedicated module that creates object proposals directly from the feature map. It utilizes a sliding window mechanism to generate a set of anchor boxes on the feature map. These anchor boxes are then refined through regression, using predefined categories and positions, to generate candidate object boxes. The RPN, thus, plays a key role in identifying potential object regions within the pseudo-image, enabling more accurate localization and classification in later stages of the network.

## 5. Backbone Network and Detection Head Modules

### 5.1. Backbone Network Module

By integrating SeNet with RPN, the backbone network is able to enhance feature representation while effectively generating candidate object regions, ultimately improving the overall performance of the 3D object detection model.

#### 5.1.1. Squeeze-And-Excitation Network (SeNet)

SeNet [36] is a channel attention module that emphasizes the significance of each feature channel, enabling the model to differentiate between crucial and less relevant channels. By amplifying important features and suppressing irrelevant ones, SeNet boosts the network’s ability to extract key features needed for target detection. Integrating this attention mechanism into the model improves performance by allowing for more precise extraction of image details and features. This attention mechanism is applied to the pseudo-images generated by the PointPillars framework, facilitating feature learning and attention weight computation. The resulting weighted features are then mapped back to the original point cloud data, enhancing both the accuracy and robustness of object detection. By combining the strengths of PointPillars with SeNet, the model generates more expressive and generalized feature representations, further improving detection capabilities.

In the previous section, a feature map M with dimensions H×W×C is generated. This feature map is subsequently processed and transformed. Global average pooling is applied to the feature map M, compressing the height and width dimensions into a vector of size 1×1×c while retaining the channel information. This vector is denoted as *z*. The *c*-th element of *z* is derived by averaging all pixel values in the *c*-th channel of the feature map M, which can be expressed as follows:

(6) $$\begin{array}{c} \tau_{c}=F_{1}\left(\lambda_{c}\right)=\frac{1}{H\times W}\sum_{i=1}^{H}\sum_{j=1}^{W}\lambda_{c}\left(i,j\right) \end{array}$$

Here, $F_{1}\left(\cdot \right)$ represents the global average pooling operation, which is used to compress the feature map. The vector $\lambda_{c}$ is the extracted feature vector, where *i* and *j* denote the positions in the *c*-th channel of the feature map. By applying global average pooling, the input feature map is compressed into an output feature vector $\tau_{c}$, allowing the model to learn the feature weights for each channel. Subsequently, $\tau_{c}$ passes through a fully connected layer, a ReLU activation layer, and a sigmoid activation layer to complete the feature excitation process, capturing the inter-channel dependencies. The calculation is expressed as follows:

(7) $$\begin{array}{c} s=F_{2}\left(\tau ,W\right)-\gamma \left(g\left(\tau ,W\right)\right)-\gamma \left(\omega_{2}g\left(\omega_{1}\tau \right)\right) \end{array}$$

Here, $\omega_{1}$ and $\omega_{2}$ are two learnable weight matrices, $\gamma \left(\cdot \right)$ represents the sigmoid activation function, and $g\left(\cdot \right)$ denotes the ReLU activation function. The feature matrix s is obtained by weighting the feature matrix $\lambda_{c}$ with the feature matrix produced after the excitation operation. The weighting formula is given by the following:

(8) $$\begin{array}{c} \tilde{x}=F_{3}\left(\lambda_{c},\tau_{c}\right) \end{array}$$

Here, $\tilde{x}$ represents the final feature matrix obtained through the element-wise multiplication of the feature matrices $\lambda_{c}$ and $\tau_{c}$.

#### 5.1.2. Region Proposal Network (RPN)

The Region Proposal Network (RPN) processes the feature map produced in the previous step to generate potential object regions. It is designed with three convolutional layers. The first convolutional layer performs downsampling on the feature map, using a stride of 2, which reduces the spatial dimensions by half. This is followed by another convolutional layer with a stride of 1, which focuses on feature extraction. These convolutional layers are followed by batch normalization (BN) and ReLU activation, which help enhance both the stability and the nonlinearity of the feature representations. Finally, the RPN performs multi-scale object detection by concatenating feature maps at different scales, allowing it to better detect objects of various sizes.

### 5.2. Detection Head Module

In the detection head module, the Single Shot Multibox Detector (SSD) is utilized to perform 3D object detection, delivering notable improvements in both detection speed and real-time performance. The SSD architecture enables the detection process to be completed in a single forward pass, eliminating the need for multiple stages of processing, which significantly boosts detection speed. Additionally, SSD supports a variety of detection head configurations, offering flexibility to meet the demands of different detection tasks and scenarios. This makes SSD highly adaptable and suitable for a broad range of applications.

Regarding the loss function, this work utilizes a method akin to that outlined in [15] to enhance model performance. The ground truth 3D bounding box is parameterized by (x,y,z,w,l,h,θ), where (x,y,z) represents the center position; (w,l,h) and θ denote the dimensions and orientation angle of the bounding box, respectively. The regression residual Δϱ=[Δx,Δw,Δθ] between the ground truth and the anchor box are calculated as follows:

(9) $$\left\{\begin{array}{c} \Delta x=\frac{x^{t}-x^{a}}{w^{a}},\Delta y=\frac{y^{t}-y^{a}}{d^{a}},\Delta z=\frac{z^{t}-z^{a}}{h^{a}}\\ \Delta w=\log\frac{w^{t}}{w^{a}},\Delta l=\frac{l^{t}}{l^{a}},\Delta h=\frac{h^{t}}{h^{a}}\\ \Delta \theta =\sin(\theta^{t}-\theta^{a}) \end{array}\right.$$

Here, $x^{t}$ denotes the ground truth and $x^{a}$ represents the anchor box, with $d^{a}=\sqrt{{\left(l^{a}\right)}^{2}+{\left(w^{a}\right)}^{2}}$. The location loss is as follows:

(10) $$L_{loc}=\sum_{\varrho \epsilon (x,y,z,w,l,h,\theta )}SmoothL_{1}\left(\Delta \varrho \right).$$

For the classification loss, focal loss [37] is employed:

(11) $$L_{cls}=-a{\left(1-p\right)}^{\kappa }\log p$$

Here, *p* denotes the probability of the anchor box, with the focus parameter κ and the scaling factor *a* for the focal loss set to 2 and 0.25, respectively.

Additionally, the orientation angle loss $L_{dir}$ is measured using the softmax classification loss over discretized orientations. By summing all the individual losses, the total loss function for the entire network is obtained, which is defined as follows:

(12) $$L_{tot}=\frac{1}{N_{pos}}\left(\nu_{loc}L_{loc}+\nu_{cls}L_{cls}+\nu_{dir}L_{dir}\right)$$

Here, $N_{pos}$ represents the number of positive sample anchors. The weights for the location loss, classification loss, and orientation loss are denoted as $\nu_{loc}$, $\nu_{cls}$, and $\nu_{dir}$, respectively, with values set to 2, 1, and 0.2.

## 6. Simulation Experiments and Analysis

### 6.1. Experimental Setup

All experimental results were analyzed using the official KITTI dataset evaluation metrics, covering both Bird’s Eye View (BEV) and 3D detection. The KITTI dataset categorizes data into three levels—easy, moderate, and hard—based on object size, occlusion, and truncation. The evaluation metric was Average Precision (AP), calculated based on Intersection over Union (IoU) thresholds. For cars, detection was considered successful if the IoU of the 3D bounding box reached 0.7; for pedestrians and cyclists, the IoU threshold was set to 0.5.

The experiments were conducted on the KITTI 3D object detection benchmark, with the training set consisting of 4501 samples and the validation set containing 4658 samples. In the experiments, the detection range for the point cloud was set to [0, 71] meters along the X-axis, [−41, 41] meters along the Y-axis, and [−4, 2] meters along the Z-axis, with a pillar resolution of (0.16, 0.16, 4) meters. Each pillar could contain up to 32 points, and the total number of pillars was capped at 16,000. During feature encoding, the dimensions of point features and pillar features were both set to 32. The proposed method was implemented using the PyTorch (version 1.10.0) framework and trained on an NVIDIA GTX 4090 platform. The training spanned 160 epochs, with an initial learning rate of 0.001, which was reduced by a factor of 0.6 every 20 epochs.

### 6.2. Comparative Analysis of Different Detection Methods

This section presents the detection results of our method, S2*-ODM, based on the S2-PFE and SeNet backbone network on the KITTI dataset. The method is further evaluated through quantitative and qualitative analyses.

#### 6.2.1. Quantitative Analysis

The detection outcomes were evaluated using the official KITTI metrics for both Bird’s Eye View (BEV) and 3D object detection. Average Precision (AP) was computed using an IoU threshold of 0.7 for cars and 0.5 for pedestrians and cyclists. The proposed method outperformed existing algorithms, as shown in Table 1 and Table 2, which compare results for BEV and 3D detection, respectively. For the moderate-difficulty level, our approach achieved BEV MAP scores of 85.46%, 53.75%, and 65.37% for cars, pedestrians, and cyclists, respectively, and 3D MAP scores of 76.38%, 61.59%, and 47.60% for these categories. Notably, the accuracy improvement in moderate 3D detection compared to PointPillars was 1.04%, 2.17%, and 3.72% for cars, pedestrians, and cyclists, respectively. These improvements were mainly due to the S2-PFE module’s ability to capture the relationship between individual pillars and the overall point cloud, combined with the effective application of the SeNet backbone network.

Moreover, despite the overall performance improvement, the accuracy gains for pedestrians and cyclists remained limited at the moderate-difficulty level (2.17% and 3.72%, respectively). This is primarily because small objects like pedestrians and cyclists have sparse point cloud data, especially at long distances, leading to incomplete geometric representations and challenging feature extraction. While the S2-PFE module improves intra-pillar relationships, it still struggles to capture subtle structures of small objects, such as limbs or bicycle frames. The SeNet backbone’s attention mechanism is less sensitive to sparse activations of small objects, resulting in lower weights assigned to relevant pillars and suppressing discriminative features. Additionally, the anchor design optimized for vehicles is ill-suited for smaller, variably oriented objects like pedestrians and cyclists, reducing the RPN’s ability to generate high-quality proposals. Finally, the imbalance in the KITTI dataset, with far fewer pedestrian and cyclist instances, biases the model towards prioritizing vehicle detection during training.

This study systematically analyzes the balance between accuracy and efficiency in 3D object detection, as well as its engineering applicability. Specifically, the evaluation results of the 3D object detection performance on the KITTI dataset are shown in Table 3, with the testing configuration being an NVIDIA GTX 4090 GPU and an Intel i9-13900K CPU. The study draws the following conclusions: in terms of the trade-off between accuracy and speed, PointPillars (23.6 FPS), which is based on pillar encoding, and the proposed S2*-ODM (20.1 FPS) in this paper demonstrate excellent real-time performance on embedded platforms. Meanwhile, HDNet (76.12% AP) and S2*-ODM (76.38% AP), with their multi-scale feature fusion mechanisms, achieve higher detection accuracy in complex scenarios. From the perspective of engineering deployment, lightweight models with fewer than 10 M parameters (such as PointPillars and S2*-ODM) are more suitable for edge computing devices.

#### 6.2.2. Qualitative Analysis

**Figure 3:**
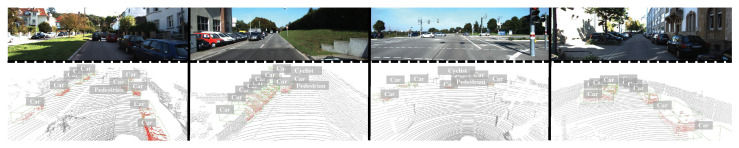
Qualitative analysis of 3D object detection performance based on the KITTI validation dataset.

**Figure 4:**
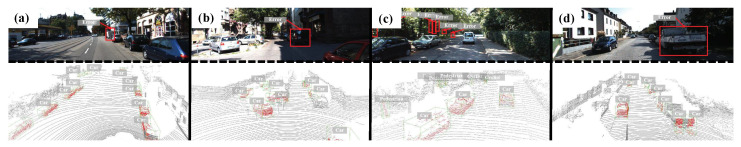
Error samples of 3D object detection based on the KITTI validation dataset. (**a**) Missed detections due to dataset annotation limitations, (**b**) Missed detections for small or heavily occluded objects, (**c**) Misclassification of vertically oriented objects, (**d**) Over-segmentation issue in object detection.

To analyze the proposed detection method qualitatively, the results are presented through 3D visualizations in Figure 3 and Figure 4. In Figure 3, the method shows strong performance in accurately detecting objects across a variety of complex scenarios, including long distances, occlusions, and overlapping objects. This success is primarily due to the model’s ability to capture both global and local features, which allows it to maintain a high detection accuracy even in challenging environments.

However, Figure 4 highlights certain limitations. First, the missed detections of certain object categories in Figure 4a (e.g., trucks) are due to the limitations of the dataset annotation protocol, which fails to provide labels for these classes and, thus, prevents the model from learning discriminative features for them. Second, Figure 4b shows missed detections for small or heavily occluded objects, mainly because of sparse point clouds (e.g., fewer LiDAR points generated by distant pedestrians) and the disruption of geometric continuity caused by occlusion, making it difficult for the model to extract effective features. Additionally, in Figure 4c, vertically oriented objects (such as poles) are misclassified as pedestrians or cyclists because of their geometric similarity to these classes in LiDAR point clouds and the model’s insufficient awareness of global scene contexts. Finally, the over-segmentation issue in Figure 4d indicates that a single object is split into multiple detections, which is likely because the pillar feature encoding fails to fully capture the relationships between adjacent pillars, especially for large or irregularly shaped objects.

### 6.3. Ablation Study Validation and Analysis

In order to evaluate the impact of different components in the proposed detection framework, a series of ablation experiments were conducted using the widely recognized KITTI validation dataset. This dataset is commonly used in the 3D object detection community to benchmark model performance, providing a diverse set of real-world driving scenarios with varying levels of complexity, such as occlusions, different object scales, and challenging environmental conditions. For comparison purposes, PointPillars was selected as the reference baseline model, as it is a well-established method in the field known for its efficiency, effectiveness, and strong performance in point cloud-based object detection tasks. By comparing the proposed framework to this baseline, the experiments aimed to highlight the contributions of each component in enhancing detection accuracy, robustness, and system performance under a variety of challenging conditions. This comparative analysis helps to demonstrate the effectiveness of the proposed approach and its ability to address the limitations of existing models.

#### 6.3.1. Single SeNet Backbone Module

The SeNet module is specifically designed to enhance the network’s attention on important features within pseudo-images while effectively filtering out irrelevant information that may lead to performance degradation. By applying attention mechanisms, SeNet helps the network focus on the most informative features, which is particularly beneficial in complex detection tasks where distinguishing between relevant and irrelevant data is crucial for accurate object recognition. The results shown in Table 4 and Table 5 highlight the impact of incorporating the SeNet module in isolation, clearly demonstrating its contribution to the overall performance of the detection framework.

The experimental findings reveal a notable increase in detection accuracy after integrating the SeNet module. Specifically, for pedestrian detection in the BEV (Bird’s Eye View) view, the mean average precision (MAP) improves by 2.26% at the moderate-difficulty level and 1.66% at the hard-difficulty level. These improvements suggest that SeNet is particularly effective in enhancing the network’s ability to detect pedestrians under various conditions, including more challenging and cluttered scenes. This could be attributed to SeNet’s capacity to selectively highlight key features, which allows the model to better distinguish pedestrians from other objects or background noise, thus improving detection accuracy.

Similarly, for cyclist detection in the BEV view, the MAP shows an improvement of 0.87% at the moderate-difficulty level and 3.20% at the hard level. The larger improvement at the hard level suggests that SeNet is particularly beneficial in handling more difficult detection scenarios, where cyclists may be partially occluded, further away, or blending in with other objects. The SeNet module’s ability to suppress irrelevant features and emphasize crucial ones likely contributes to this enhanced performance, enabling the model to more effectively identify cyclists even in these more complex and demanding situations.

These results underscore the effectiveness of the SeNet module in refining feature extraction processes and boosting overall detection performance. By improving the model’s focus on key features and reducing the influence of irrelevant information, SeNet helps the detection framework achieve higher precision in real-world, challenging conditions. The positive impact on both pedestrian and cyclist detection further emphasizes the versatility and importance of attention mechanisms in enhancing the robustness and accuracy of 3D object detection systems.

#### 6.3.2. Single S2-PEE Module

The results presented in Table 4 and Table 5 demonstrate the substantial impact of incorporating only the S2-PEE (pillar feature encoding enhancement) module into the overall detection architecture. The addition of the S2-PEE module leads to significant improvements across a variety of performance metrics, showcasing its ability to effectively capture relational features both within individual pillars and between them. By considering not just the local features of each pillar, but also the global relationships between different pillars in the point cloud, the S2-PEE module enables the network to gain a deeper understanding of the scene and improve object detection accuracy.

Specifically, for vehicle detection in 3D, the inclusion of the S2-PEE module results in a notable 4.29% increase in accuracy at the easy-difficulty level. This improvement can be attributed to the S2-PEE module’s ability to better represent the geometric relationships between pillars, which is especially useful for detecting vehicles that are clearly visible and have less occlusion or distortion. The module’s enhanced feature encoding helps the network to more accurately locate and classify vehicles in relatively simpler scenes, where the global structure of the environment is more easily discernible.

For pedestrian detection in 3D, the accuracy improves by 2.78% at the moderate-difficulty level and 1.22% at the hard-difficulty level. The improvements at both levels suggest that the S2-PEE module is effective in handling detection tasks of varying complexities. At the moderate level, pedestrians may be more easily identifiable, but the additional relational context provided by the S2-PEE module allows the model to better distinguish pedestrians from background noise or similar objects. At the hard level, where pedestrians may be occluded, further away, or interacting with other objects, the S2-PEE module’s enhanced ability to capture relational features becomes particularly beneficial, allowing the model to accurately detect pedestrians despite these challenges.

Similarly, for cyclist detection in 3D, a 0.96% improvement is observed at the hard-difficulty level. This improvement, though smaller in comparison to vehicle and pedestrian detection, is still significant, especially considering the challenges posed by cyclist detection in complex environments. Cyclists can often be smaller, partially occluded, or moving in unpredictable ways, which makes their detection more difficult. The S2-PEE module helps the model to better understand the spatial relationships between different pillars, allowing it to more effectively detect cyclists even in challenging situations.

These results highlight the effectiveness of the S2-PEE module in enhancing detection performance across different object categories and difficulty levels. By improving the ability to capture both local and global relational features, the S2-PEE module enables the detection framework to achieve a higher accuracy, particularly in more complex or occluded environments. The improvements in detection accuracy for vehicles, pedestrians, and cyclists demonstrate the versatility of the S2-PEE module, making it a valuable component for improving the robustness and precision of 3D object detection systems.

#### 6.3.3. S2-PEE Combined with SeNet Module

The results shown in Table 4 and Table 5 highlight the significant improvements in detection performance achieved by combining the two modules, S2-PEE and SeNet. The integrated approach consistently outperforms the baseline model and even surpasses the scenarios where only one of the modules is utilized in isolation. This clearly demonstrates the powerful synergistic effect of the S2-PEE and SeNet backbone modules in boosting overall model performance. The integration of both modules allows the system to capture more nuanced and complex features from the point cloud data, thus improving the model’s ability to detect objects with higher precision and accuracy.

**Figure 5:**
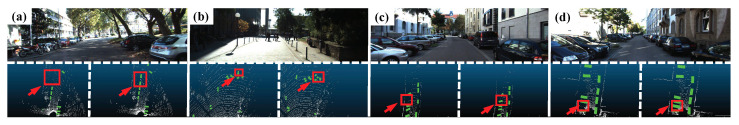
Comparison of the detection method based on a single S2-PFE module with the baseline PointPillars, showing the effects before and after improvements in 3D object detection. (**a**,**c**,**d**) demonstrate improvements in addressing missed detection issues, while (**b**) highlights improvements in addressing under-segmentation issues.

To further assess the impact of the proposed method, visualized results are provided in Figure 5, offering a clear and intuitive illustration of the effects brought about by the incorporation of the S2-PEE and SeNet modules. The left side of Figure 5a–d showcases the limitations of the baseline model, particularly in terms of its ability to handle the relationships between individual pillars and the broader context of the point cloud. In challenging scenarios, such as those involving object overlap or occlusion, the baseline model often struggles with under-segmentation and missed detections. The failure to account for the intricate interactions between pillars and the surrounding point cloud results in incomplete or inaccurate object detection, which can be detrimental to applications like autonomous driving, where precise object localization is critical.

In contrast, the enhanced method that incorporates the S2-PEE module significantly improves the model’s ability to capture these relationships between pillars. By enhancing the feature encoding process, the S2-PEE module helps the network to better understand the spatial relationships between objects in the scene, leading to a significant reduction in false detections and under-segmentation. The integration of SeNet further refines the focus of the model by emphasizing relevant features, further minimizing irrelevant background noise. This combined effect results in more accurate and reliable object detection, even in scenarios where the point cloud data are noisy or incomplete.

The right side of Figure 5a–d highlights the enhanced capability of the modified network to detect occluded objects. In these cases, where parts of objects may be hidden or obscured by other structures, the proposed method demonstrates a remarkable improvement in detection accuracy. The system is able to better infer the presence of occluded objects, offering more complete and precise object localization. This is particularly important in real-world driving environments, where occlusions are common due to the presence of other vehicles, pedestrians, and environmental obstacles.

These results, presented from a BEV (Bird’s Eye View) perspective, emphasize the key improvements made by the proposed method. The BEV perspective is particularly useful for visualizing 3D object detection results as it provides a clear top-down view of the scene, allowing for easy comparison of the performance of the baseline and enhanced models. By combining the strengths of the S2-PEE and SeNet modules, the proposed method is able to achieve superior performance in detecting objects, even in challenging conditions such as occlusion, overlap, and varying object scales.

In summary, the visualized results not only demonstrate the effectiveness of the integrated approach but also provide valuable insights into the specific improvements achieved by the incorporation of the S2-PEE and SeNet modules. The enhanced detection capability, particularly in challenging scenarios, underscores the potential of this method to improve the robustness and reliability of 3D object detection systems in real-world applications.

## 7. Conclusions and Future Work

This paper introduces an enhanced pillar-based 3D object detection method, S2*-ODM, which integrates a dual-stage pillar feature encoding (S2-PFE) module with a SeNet-augmented backbone network. The S2-PFE module, as a crucial component within the pillar-based 3D detection framework, substantially improves the network’s capacity to handle challenging detection scenarios, particularly those involving occlusion or overlapping objects. At the same time, the incorporation of SeNet into the backbone network enables the model to concentrate more effectively on essential features by suppressing irrelevant ones. Experimental results on the KITTI dataset show that the proposed method outperforms existing detection techniques in terms of accuracy. Specifically, in moderate-difficulty conditions, the 3D detection average precision (AP) for vehicles, pedestrians, and cyclists increased by 1.04%, 2.17%, and 3.72%, respectively, when compared to the baseline model. Additionally, ablation studies confirm that the S2-PFE module plays a pivotal role in enhancing detection performance, further validating the effectiveness of the proposed method.

The qualitative evaluation further indicates that the proposed method reduces under-segmentation and missed detections in occluded or overlapping scenarios. However, the method lacks an assessment of varying degrees of occlusion and overlap, necessitating further validation in diverse occlusion scenarios.

To further enhance detection performance, future research may consider integrating multimodal data. We plan to enhance our model’s generalization capability across diverse scenarios through multi-dataset validation (e.g., nuScenes and Waymo Open Dataset), synthetic data augmentation using CARLA-SIM to simulate complex weather and lighting conditions, dynamic occlusion handling with a density-aware attention module, and multi-sensor fusion by integrating thermal camera and radar data. In addition, we will develop an adaptive feature weighting mechanism to prioritize visible regions in heavily occluded scenes, and we will quantify detection confidence to filter out low-reliability predictions, thereby reducing false positives.

## 8. Abbreviation Explanation

This section provides explanations for some of the abbreviations used in the paper, as shown in Table 6.

## Figures and Tables

**Table 1:** Comparison of BEV detection accuracy for different algorithms on the KITTI test set. Red indicates the lowest value, while green indicates the highest value.

Method	Car (%)			Pedestrian (%)			Cyclist (%)		
	**Easy**	**Mod.**	**Hard**	**Easy**	**Mod.**	**Hard**	**Easy**	**Mod.**	**Hard**
PointPillars [15]	88.44	86.61	80.34	59.17	50.74	47.70	79.65	62.76	56.51
SECOND [19]	88.14	79.44	78.02	55.17	46.34	44.83	73.74	56.11	48.85
VoxelNet [13]	89.71	79.62	77.75	46.49	41.10	38.47	67.06	55.12	50.91
AVODFPN [36]	88.60	83.86	77.97	58.82	51.57	47.61	68.16	57.55	50.84
FPointNet [38]	89.17	84.47	75.80	58.56	50.69	47.67	75.85	62.43	55.15
HDNET [37]	89.58	87.01	78.76	N/A	N/A	N/A	N/A	N/A	N/A
PRGBNet [39]	91.46	85.80	80.75	38.14	29.39	27.01	73.16	57.66	51.85
TANet [40]	91.79	86.75	81.40	60.01	51.59	47.75	79.37	63.98	56.42
S2*-ODM	90.13	85.46	80.04	60.79	53.75	49.64	83.58	65.37	61.25

**Table 2:** Comparison of 3D detection accuracy for different algorithms on the KITTI test set. Red indicates the lowest value, while green indicates the highest value.

Method	Car (%)			Pedestrian (%)			Cyclist (%)		
	**Easy**	**Mod.**	**Hard**	**Easy**	**Mod.**	**Hard**	**Easy**	**Mod.**	**Hard**
PointPillars [15]	79.40	75.34	68.65	76.13	59.42	53.27	52.43	43.88	41.84
SECOND [19]	83.36	73.89	66.43	70.74	54.08	47.13	51.30	42.79	37.52
VoxelNet [13]	77.71	65.35	57.97	61.46	48.60	44.61	39.72	33.93	31.74
AVODFPN [36]	82.32	72.26	66.76	64.38	52.56	46.99	51.18	43.19	41.26
FPointNet [38]	81.64	70.83	62.63	72.40	57.21	50.83	51.65	45.33	40.67
HDNET [37]	83.28	76.12	71.25	75.32	61.78	55.40	44.71	37.45	35.00
PRGBNet [39]	84.31	73.81	68.88	67.37	52.47	47.10	35.09	26.72	24.35
TANet [40]	84.58	76.13	69.01	75.89	59.63	52.72	53.91	44.53	40.68
S2*-ODM	83.81	76.38	74.17	81.52	61.59	57.86	54.28	47.60	43.27

**Table 3:** Comparison of 3D detection computational performance for different algorithms on the KITTI test set.

Method	KITTI AP (Car, Moderate %)	Model Parameter Count (M)	FPS
PointPillars [15]	75.34	4.8	23.6
SECOND [19]	73.89	6.2	18.1
VoxelNet [13]	65.35	9.1	14.5
AVOD-FPN [36]	72.26	12.4	10.2
FPointNet [38]	70.83	7.9	8.7
HDNet [37]	76.12	15.3	6.5
PRGBNet [39]	73.81	5.7	16.8
TANet [40]	76.13	8.4	12.3
S2*-ODM	**76.38**	**5.6**	**20.1**

**Table 4:** Comparison of BEV detection accuracy on the KITTI test set with different modules ablated. Red indicates the lowest value, while green indicates the highest value.

Method	Car (%)			Pedestrian (%)			Cyclist (%)		
	**Easy**	**Mod.**	**Hard**	**Easy**	**Mod.**	**Hard**	**Easy**	**Mod.**	**Hard**
PointPillars [15]	88.62	86.37	80.10	58.93	50.50	47.46	79.41	62.52	56.27
Single SeNet	90.31	86.63	80.11	58.49	52.76	49.12	79.65	63.39	59.47
Single S2-PFE	90.00	85.29	80.24	58.79	53.01	49.51	81.29	62.21	59.77
**With both**	89.96	86.93	80.87	59.84	53.58	49.47	83.41	65.20	61.08

**Table 5:** Comparison of 3D detection accuracy on the KITTI test set with different modules ablated. Red indicates the lowest value, while green indicates the highest value.

Method	Car (%)			Pedestrian (%)			Cyclist (%)		
	**Easy**	**Mod.**	**Hard**	**Easy**	**Mod.**	**Hard**	**Easy**	**Mod.**	**Hard**
PointPillars [15]	79.46	75.40	68.71	52.49	43.94	41.90	76.19	59.48	53.33
Single SeNet	84.33	76.57	69.54	52.50	47.45	43.02	76.76	59.62	55.99
Single S2-PFE	83.49	75.87	69.03	52.77	46.72	43.12	76.11	58.41	54.29
**With both**	83.75	76.32	74.11	54.22	47.54	43.21	81.46	61.53	57.80

**Table 6:** Abbreviation explanation.

Abbreviation	Explanation
**KITTI**	A widely used public dataset in the field of autonomous driving, covering tasks such as 3D object detection and optical flow estimation
**BEV**	Bird’s Eye View perspective, which is used to evaluate 3D detection results from a top-down view
**S2-PFE**	Dual-Stage Pillar Feature Encoding: A dual-stage pillar feature encoding module that captures intricate relationships within and between pillars to enhance object differentiation
**SeNet**	Squeeze-and-Excitation Network: A channel attention network that recalibrates channel-wise feature responses adaptively to improve feature extraction
**RPN**	Region Proposal Network: A network module that generates region proposals for object detection tasks, enhancing the detection of critical features in pseudo-images

## Data Availability

Dataset available on request from the authors.
